# Cannabidiol in Sports: A Brazilian Perspective

**DOI:** 10.1021/acsptsci.5c00580

**Published:** 2025-10-09

**Authors:** Tatyana Nery, Jose Wilson NV Andrade, Jimmy Fardin Rocha, Ana Paula Pinto de Araújo, Aderbal Silva Aguiar Jr

**Affiliations:** † LABIOEX-Laboratory of Exercise Biology, 28117 Federal University of Santa Catarina-UFSC, Araranguá, Santa Catarina 88905-120, Brazil; ‡ Institute for Education and Research (IEP) Remederi, São Paulo, São Paulo 04149-120, Brazil

**Keywords:** cannabidiol, sports medicine, recovery, antidoping

## Abstract

This Viewpoint examines the emerging role of cannabidiol (CBD) in sports medicine, with a particular emphasis on its potential to support athlete health through indirect mechanisms. Rather than acting as a direct performance enhancer, CBD may contribute to improved readiness for training and competition by promoting better sleep, alleviating anxiety, and accelerating recovery. We also discuss challenges for antidoping compliance, including product contamination and regulatory gaps in Brazil, and highlight CBD’s promise as a safer alternative to opioids for pain management in athletes.

## Graphical abstract

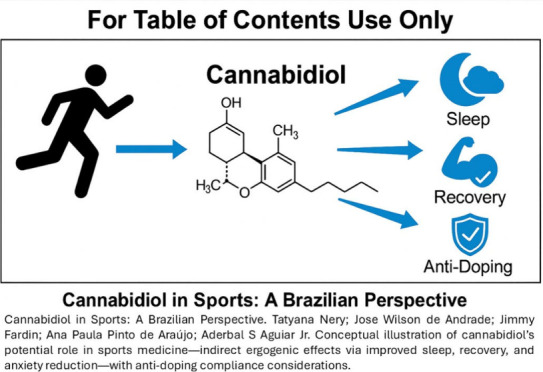

## Shifting Paradigms in Sports Medicine: Cannabidiol’s Emerging Role from a Brazilian Perspective

Sports medicine is evolving rapidly, creating opportunities to integrate novel therapeutic strategies. We hypothesize that cannabidiol (CBD), though not a direct performance enhancer, may improve athletic readiness and training availability through its effects on sleep, anxiety, and recovery—an indirect ergogenic pathway that warrants athlete-specific investigation. In 2018, the World Anti-Doping Agency (WADA) formally excluded CBD from its Prohibited List, creating new opportunities for its exploration in athletic settings. Since then, international and Brazilian athletes have shown increasing interest in its potential applications, motivated both by therapeutic expectations and by the need for safer alternatives to conventional pharmacological strategies..

Historically, Brazil’s cultural and regulatory environment has been conservative, maintaining strict controls over cannabis-derived products. Recent changes implemented by the Brazilian Health Regulatory Agency (ANVISA) have created new pathways for access, although barriers remain compared with other countries.

CBD use among athletes has increased since WADA’s policy change. This trend reflects both a shifting regulatory landscape and a growing body of research suggesting potential benefits for athletic populations. Recent studies indicate that one-third of endurance athletes report using cannabinoids, with usage rates increasing with age and reaching a prevalence of 28% in contact sports such as rugby.^1–3^

This Viewpoint examines the current evidence on medical cannabis in sports, focusing specifically on CBD from a Brazilian perspective. We explore its pharmacological basis, potential benefits for athletes, quality control challenges, and ethical considerations. Recognizing the limitations of existing research, we propose that CBD’s effects on sleep quality, muscle recovery, and anxiety reduction may indirectly improve athletic performance, suggesting a potential ergogenic effect that warrants further investigation.

## The Ergogenic Potential: A New Perspective

Although the current literature does not explicitly classify CBD as an ergogenic aid, we propose that its effects on recovery, sleep, and anxiety may indirectly enhance performance, thereby constituting an ergogenic effect. This perspective represents a paradigm shift in sports medicine, expanding the concept of ergogenic aids beyond direct physiological enhancement during exercise.

Sleep restriction impairs cognitive and physical performance; by improving sleep architecture and reducing disturbances, CBD may therefore support recovery processes and enhance readiness for training and competition, although studies in athletes remain limited.^4,5^

Collectively, these effects—improved sleep quality, reduced anxiety, and enhanced recovery—create conditions conducive to improved performance, suggesting an indirect ergogenic effect that warrants further scientific investigation.

## Quality Control and Antidoping Considerations

While CBD is permitted by WADA, all other cannabinoids remain prohibited; in-competition AAFs for THC are triggered when urinary carboxy-THC exceeds 150 ng/mL (DL 180 ng/mL).^13^

Despite CBD’s removal from the World Anti-Doping Agency (WADA) Prohibited List, quality control challenges remain and pose significant risks for athletes. Studies have shown that commercially available CBD products may contain THC and other prohibited cannabinoids at levels capable of producing positive antidoping test results.^6–8^ A comprehensive analysis found that 4 of 11 CBD products contained THC concentrations sufficient to yield urinary levels exceeding WADA’s threshold.^8^ Although CBD itself is permitted by WADA, the principle of strict liability continues to pose a critical challenge. Athletes remain fully accountable for any prohibited substances detected in their samples, regardless of intent. This responsibility becomes problematic when CBD products contain undeclared cannabinoids such as THC, as documented in several market analyses.

Brazil’s regulatory environment adds another layer of complexity. While medical cannabis has been approved by the Brazilian Health Regulatory Agency (ANVISA) for specific conditions, the regulatory framework for CBD products remains evolving and complex. Resolution RDC No. 327/2019 ^9^ established specific regulations for cannabis-derived products, but the approval process remains rigorous and limited to certain medical indications. This regulatory uncertainty complicates access to safe and legal CBD products for athletes.^10^, This regulation is currently under public review in 2025, which may change quality requirements and access pathways.

Manufacturing standards and quality control for CBD products vary considerably, creating additional risks. Factors such as extraction methods, formulation processes, and storage conditions can affect product composition and stability. Without standardized manufacturing practices and rigorous quality control, athletes cannot rely on product consistency or safety.

To safeguard athletes, robust quality control measures are essential. These include third-party testing for prohibited substances, transparent labeling of all ingredients, and certification programs tailored to sports supplements. Education initiatives are also critical to inform athletes about these risks and encourage the use of products verified to be free of prohibited substances.

## An Alternative to Opioids: Addressing a Growing Concern

As tramadol became prohibited in-competition as of January 1, 2024, interest in nonopioid strategies—including CBD—has grown within sports medicine.^13^

Frequent use of analgesics and opioids among athletes represents a significant public health concern. Studies indicate that athletes across multiple sports regularly use prescription and over-the-counter pain medications, often exceeding recommended dosages.^8^ A systematic review highlighted troubling patterns of analgesic use among elite athletes, including self-medication, inadequate medical supervision, and long-term health consequences.^8^ Such practices carry substantial risks, including dependence, gastrointestinal complications, cardiovascular effects, and impaired recovery processes.

In Brazil, where access to specialized sports medicine care varies by region and competition level, inadequate pain management strategies can exacerbate this problem. Limited access to multidisciplinary approaches may lead athletes to rely heavily on pharmacological interventions, frequently without proper medical oversight. Cultural factors, including the normalization of pain in sports and pressure to perform despite injury, further contribute to problematic analgesic use.

CBD presents a potential alternative to traditional pain management strategies in sports. Preliminary evidence suggests that CBD may exert analgesic effects through modulation of inflammatory processes and interaction with pain signaling pathways.^11,12^ Unlike opioids, CBD has not been associated with dependence or severe adverse effects, making it a potentially safer option for long-term pain management in athletic populations.

The potential for CBD to reduce reliance on nonsteroidal anti-inflammatory drugs (NSAIDs) is particularly relevant for endurance athletes and those participating in sports requiring frequent training sessions. Prolonged NSAID use has been linked to gastrointestinal complications, renal dysfunction, and impaired muscle adaptation. If CBD can effectively manage pain and inflammation with fewer adverse effects, it could serve as a valuable alternative or adjunct to traditional analgesics in appropriate clinical contexts.

## Future Directions and Recommendations

Advancing our understanding of CBD’s role in sports medicine requires addressing several key areas. First, athlete-specific research is essential to determine optimal dosing, timing, and administration routes for targeted outcomes. Current evidence is largely derived from nonathletic populations or preclinical models, limiting applicability to competitive sports. Studies examining CBD’s effects across different training modalities, competition demands, and recovery protocols would provide valuable insights for evidence-based implementation.

Second, standardization of CBD products for sports use is crucial. This includes developing sport-specific quality standards, testing protocols, and certification programs that address the unique needs and regulatory requirements of athletes. Such standardization would reduce contamination risks and increase athlete confidence in product safety. In Brazil, this could involve collaboration between ANVISA, sports organizations, and pharmaceutical companies to create athlete-focused CBD formulations with rigorous quality control.

Third, evidence-based guidelines for CBD use in sports medicine are needed. These should address indications, contraindications, potential interactions with other supplements or medications, and recommendations tailored to specific sports and competition levels. Developing these guidelines will require collaboration among sports medicine professionals, researchers, regulatory bodies, and athlete representatives.

Finally, educational programs for athletes, coaches, and healthcare providers are essential. These should deliver accurate information on CBD’s benefits, limitations, legal status, and quality control considerations, enabling informed decision-making regarding its use in sports settings.

## Conclusions

From a Brazilian standpoint, CBD represents a promising addition to sports medicine, with potential benefits for recovery, anxiety, and pain management that may indirectly enhance performance. Its implementation, however, requires navigating unresolved challenges: product quality control, regulatory uncertainties, and the scarcity of athlete-specific evidence. Addressing these barriers will depend on collaborative efforts between researchers, sports organizations, and regulatory agencies. Ultimately, Brazil offers a unique setting to develop evidence-based models that integrate CBD into athlete care while safeguarding fairness and integrity in competition.

Significant challenges remain, including quality control issues, regulatory uncertainties, and the limited amount of athlete-specific research. Addressing these barriers will require collaborative efforts among researchers, regulatory agencies, sports organizations, and healthcare professionals. In Brazil, evolving regulations, cultural factors, and uneven access to sports medicine resources create a unique environment for CBD implementation.

From a Brazilian perspective, CBD offers an opportunity to develop evidence-based strategies that promote athlete health and performance while respecting competitive integrity. Achieving this goal will require not only scientific advancement but also regulatory innovation, targeted education, and cultural shifts in attitudes toward cannabis-derived medicines in sports contexts.
